# Current status of human adenovirus infection in China

**DOI:** 10.1007/s12519-022-00568-8

**Published:** 2022-06-18

**Authors:** Nai-Ying Mao, Zhen Zhu, Yan Zhang, Wen-Bo Xu

**Affiliations:** NHC Key Laboratory of Medical Virology and Viral Diseases, WHO WPRO Regional Reference Laboratory of Measles and Rubella, Chinese Center for Disease Control and Prevention, Measles Laboratory in National Institute for Viral Disease Control and Prevention, 155# Changbai Road, Changping District, Beijing, 102206 China

**Keywords:** Acute respiratory tract infections, Conjunctivitis, Gastroenteritis, Human adenovirus, Hepatitis, Unknown etiology

## Abstract

**Background:**

Outbreaks of severe, acute hepatitis among children have recently attracted global attention. The pathogen causing the outbreak remains unknown, but there is growing evidence that it may be associated with human adenovirus (HAdV).

**Data sources:**

A review of adenovirus-related clinical studies, epidemiological studies, etiological studies, and case reports was conducted by reviewers independently.

**Results:**

HAdV can cause a wide variety of clinical symptoms. In the Mainland of China, HAdV infection accounts for 5.8%–13% of patients with acute respiratory infections, and these infections are mainly caused by species B, C, and E of HAdV. For acute conjunctivitis, 39.8%–74.9% of sporadic cases were infected by B and D species of HAdV. Outbreaks of keratoconjunctivitis and pharyngoconjunctival fever related to HAdV infection could be found throughout the country. In pediatric patients with acute gastroenteritis, HAdV-41 was the predominant HAdV type, followed by HAdV species B and C. Several types of HAdV, including HAdV-5, HAdV-7, HAdV-1, and HAdV-2, have previously been reported as potential pathogens associated with HAdV hepatitis in immunocompromised patients. However, few HAdV-related hepatitis cases have been reported in China to date.

**Conclusions:**

There are no systematic surveillance and clinical studies on HAdV hepatitis in China. Therefore, it is imperative to establish a nationwide HAdV virological surveillance system to collect relevant clinical, epidemiological and virological surveillance data and risk factor information as soon as possible to assess the potential risk of HAdV hepatitis among children.

## Introduction

Human adenovirus (HAdV) is a highly contagious pathogen that can cause a wide variety of clinical symptoms, including respiratory tract disease, conjunctivitis, gastroenteritis, and urinary tract infection [1–4]. This infection is generally self-limiting; however, in immunocompromised patients, it can lead to severe damage to multiple organs, such as the liver, heart, meninges, and brain [5]. HAdV belongs to the family *Adenoviridae* within the genus *mastadenovirus*, with a nonenveloped, icosahedral viral particle. HAdV contains a double-stranded DNA genome, ranging from 26 to 45 kb in length [6]. To date, HAdVs can be divided into seven species (A-G), with at least 113 types (http://www.hadvwg.gmu.edu). Most novel HAdVs originate from intertypic recombination, which could result in changes in viral fitness, tissue tropism, and virulence, such as HAdV-55 and HAdV-56 [7–9]. As of April 2022, the World Health Organization has reported severe acute hepatitis outbreaks among children from 11 countries across Europe and America, which have induced great public concern. Of the 169 reported hepatitis cases, HAdV was detected in at least 74 cases (43.8%), and 18 were identified as HAdV-41 belonging to the F species. Accordingly, HAdV is currently presumed to be a possible etiology of this outbreak [10]. Therefore, the aim of this review was to summarize the epidemiological and genetic characteristics of HAdV circulating in China, as well as the relationship between severe liver function impairment and HAdV infections.

## Acute respiratory tract infections

Multiple HAdV species are commonly associated with acute respiratory infections (ARIs), including species B, C, and E [11–14]. In the Mainland of China, several studies have shown that HAdV accounts for 5.8%–13% of patients with ARIs, depending on different enrollment criteria, such as geographical and climatic factors. Children under 5 years of age were reported to be most susceptible to HAdV infection [15–18]. HAdV has been detected at a significantly higher rate in hospitalized children and young adults than in outpatients, suggesting that it is a predictor of the severity of ARIs. Of these, HAdV-7 and HAdV-3 are the predominant types identified in patients with ARIs, although the detected types may change over time and geography [17]. In contrast, HAdV-7 infection in children tends to have more severe clinical consequences than HAdV-3 [19–21]. During the past few decades, outbreaks of pediatric lower respiratory infections caused by HAdV-7 have been reported in China [22–24].

In addition, HAdV-55, which belongs to species B, plays an important role in ARIs. Since it was first isolated from Shaanxi Province in 2006, HAdV-55 has spread widely and is causing continuous outbreaks in China. Based on retrospective surveillance data, HAdV-55 was identified in more than half of the provinces throughout China during the period between 2006 and 2016 [25]. Furthermore, HAdV-55 has gradually become the leading cause of community-acquired pneumonia (CAP) in China [26]. A retrospective study that enrolled 969 CAP cases showed that HAdV-55 accounted for 43.75% (21/48) of HAdV-related CAP cases in Beijing and Yantai among the adolescent and adult populations during the period 2010–2012 [27]. ARI outbreaks related to HAdV-55 have frequently been reported in China, especially in military camps. In 2016, three outbreaks in military camps occurred successively in Tibet, Sichuan, and Yunnan Provinces, and more than 300 infections were recorded [28].

Although species C of HAdV (HAdV-C) is generally considered a common pathogen in pediatric patients with ARIs, the leading contributor of disease in the population of China is still unclear. HAdV-C infection is usually self-limited but could lead to severe consequences in immunocompromised hosts, such as transplant recipients [29]. To date, four novel types of HAdV-C have been formally recognized, namely, HAdV-57, 89, 104, and 108, two of which have originated from China. However, a retrospective study from 2000 to 2016 showed that 16 new genetic patterns of HAdV-C were identified from nine provinces, covering six administrative regions. These results indicated that multiple potential recombinant strains of HAdV-C are currently cocirculating in China [30].

## Conjunctivitis

Generally, species B and D of HAdV are responsible for ocular infections, including keratoconjunctivitis (EKC), pharyngoconjunctival fever (PCF), and acute hemorrhagic conjunctivitis (AHC) [31]. However, different types of HAdV are associated with distinct clinical symptoms, age, and underlying medical conditions. Surveillance conducted in 18 hospitals in Beijing during the period 2011–2013 showed that HAdV was responsible for 39.8% of the acute conjunctivitis cases. Fifteen types of HAdV were identified among adenoviral conjunctivitis cases. Among these types, five (HAdV-4, 37, 53, 64, and 8) accounted for 81.1% of adenoviral conjunctivitis cases [32]. Another study showed that 74.86% of conjunctivitis cases were caused by seven types of HAdV in Jiangxi Province during the period 2011–2012. Among them, HAdV-7 (47.82%), HAdV-3 (30.43%), and HAdV-55 (8.70%) were the most common types, followed by HAdV-4 (8.70%), HAdV-21 (1.45%), HAdV-37 (1.45%), and HAdV-64 (1.45%) [33].

In addition to sporadic cases, adenoviral conjunctivitis is associated with disease outbreaks. During EKC outbreaks, HAdV-8, 19, 37, and 54 were detected frequently, with HAdV-8 being the predominant type over time and geographical location. To date, three EKC outbreaks caused by HAdV have been reported in Liaoning, Tibet, and Yunnan Provinces in China successively during 2012–2017, and HAdV-8 and HAdV-56 were identified as the etiologies for these outbreaks [2, 34, 35]. PCF outbreaks were associated with waterborne transmission of various adenovirus types, such as HAdV-3, HAdV-4, and HAdV-7, with HAdV-3 being the most common etiological agent. Several outbreaks of PCF in swimming pools caused by HAdV-3 and HAdV-7 have been reported in China [36–38]. Global data indicate that AHC outbreaks are caused mainly by HAdV-2, 7, 8, and 11, which are usually coinfected with human enterovirus [39], whereas few AHC outbreaks related to HAdV have been reported in China to date.

## Gastroenteritis

Rotavirus and norovirus are leading causes of viral gastroenteritis among children and adults worldwide. HAdV-related gastroenteritis occurs most often in children younger than 4 years of age [6, 40]. In HAdV-associated viral diarrheal disease, HAdV species F, including HAdV-40 and HAdV-41, were the most frequently detected, while species A, C, and D have also been reported [41]. A study from Shandong Province during 2017–2018 showed that the detection rate of HAdV was 7.47% among 656 enrolled fecal specimens, of which seven types of HAdV within four species (A, B, C, and F) were identified. Among them, HAdV-41 (48.98%) was the most common, followed by HAdV-3, HAdV-31, HAdV-7, HAdV-40, HAdV-1, and HAdV-2 [42]. Similar results were observed in a study conducted in Shanghai city during the period 2010–2011, showing that HAdV, with a 7.1% detection rate, was the second most frequently detected pathogen after rotavirus infection among pediatric patients with acute gastroenteritis and that HAdV-41 was the predominant HAdV type [43]. However, another study showed that HAdV-3 was most frequently detected in acute gastroenteritis cases in children under 5 years of age in Fujian Province from 2009 to 2017, followed by HAdV-41, 2, 1, 40, 7, and 12 [44].

## HAdV hepatitis

Hepatitis-associated HAdV infection has been reported mainly in immunosuppressed patients [5]. Severe infections involving multiple organs, including the liver, have been observed [45]. Research data from 1960 to 2012 showed that of the 89 reported HAdV hepatitis cases worldwide, 43 (48%) were liver transplant recipients, 19 (21%) were bone marrow transplant recipients, and 11 (12%) had recently received chemotherapy for malignancy [5]. Another study from Stanford University Medical Center during the period 1995–2016 showed that among 12 HAdV hepatitis cases, eight were children. Of these children, seven received liver transplantation, and one received lymphocytic leukemia chemotherapy [46]. The above studies indicate that HAdV hepatitis cases occur commonly in liver transplant recipients, and of these, approximately 65% of HAdV hepatitis cases occur in children. Studies have shown that the incidence of HAdV hepatitis in childhood liver transplant recipients was 2%–4% [47], which might be highly related to the primary infection, but the association of viral reactivation with HAdV hepatitis cases could not be excluded.

Several sporadic HAdV hepatitis cases in healthy children and adults have been reported in the European and American regions [48–50]; therefore, HAdV infection should also be considered as a possible etiology during the differential diagnosis of severe acute liver hepatitis in children. Several types of HAdV, including HAdV-5, HAdV-7, HAdV-1, and HAdV-2, which were previously reported as the primary cause of ARIs, have also been reported as potential pathogens associated with HAdV hepatitis [51–54]. However, because there is no systematic surveillance and study on HAdV hepatitis, few HAdV hepatitis cases, including immunosuppressed or healthy individuals, have been reported in China to date.

## Etiology hypotheses of the emerging severe acute hepatitis in children

The pathogen responsible for the outbreaks of pediatric severe acute hepatitis around the world remains unknown. However, several speculations have been proposed: (1) opportunistic infections caused by HAdV might occur in children with several cofactors, which could lead to severe inflammation or immunopathology in the liver. Possible cofactors include previous SARS-CoV-2 or another pathogen infection, toxin, drug or environmental exposure, and enhanced susceptibility due to lack of exposure of HAdV during the pandemic of COVID-19; (2) genetic mutation or recombination of HAdV lead to increased virulence or altered tissue tropism; (3) infected with a novel pathogen or coinfected with HAdV; (4) infected with a novel variant of SARS-CoV-2; (5) Immunization of SARS-CoV-2 mRNA vaccine could elicit T-cell-dominant autoimmune hepatitis [55], and  (6) caused by noninfectious etiology, such as drugs, toxins or environmental exposure. However, the current etiology mainly points to HAdV infection, especially HAdV-41. HAdV-41 is rarely reported to cause hepatitis, while the possibility of HAdV-41 and its variants causing liver injury cannot be ruled out. Further evidence, including etiology, genomics, liver pathology, and immunohistochemistry, is needed for confirmation.

## Conclusions

There is currently a lack of effective antiviral drugs and vaccines for the treatment and prevention of HAdV infections. It is imperative to establish nationwide HAdV virological surveillance based on clinical symptoms and to collect relevant epidemiological and virological surveillance data and risk factor information as soon as possible to assess the potential risk of HAdV hepatitis among children in China and to provide scientific and technical support for the prevention and control of HAdV-related diseases.
