# Correction to: Current status of human adenovirus infection in China

**DOI:** 10.1007/s12519-022-00587-5

**Published:** 2022-07-30

**Authors:** Nai-Ying Mao, Zhen Zhu, Yan Zhang, Wen-Bo Xu

**Affiliations:** NHC Key Laboratory of Medical Virology and Viral Diseases, WHO WPRO Regional Reference Laboratory of Measles and Rubella, Chinese Center for Disease Control and Prevention, Measles Laboratory in National Institute for Viral Disease Control and Prevention, 155# Changbai Road, Changping District, Beijing, 102206 China

## Correction to: World Journal of Pediatrics 10.1007/s12519-022-00568-8

In the original publication, the author has missed to include the reference 20 “Fu Y, Tang Z, Ye Z, Mo S, Tian X, Ni K, et al. Human adenovirus type 7 infection causes a more severe disease than type 3. BMC Infect Dis. 2019;19:36” in the reference list.

The original article has been corrected.

